# CT-3D MERGE fusion imaging improves image quality compared with CT and 3D MERGE in patients with lumbar disc herniation

**DOI:** 10.3389/fneur.2024.1490033

**Published:** 2024-12-13

**Authors:** Hongyi Li, Hailong Liu, Mengqiang Xiao, Xiaodan Du, Meng Zhang, Jingzhi Ye, Ming Lei, Jun Chen, Jie Chen

**Affiliations:** ^1^Department of Radiology, Guangdong Provincial Hospital of Chinese Medicine, Zhuhai, China; ^2^Department of Medical Imaging, Zhuhai Hospital of Integrated Traditional Chinese and Western Medicine, Guangdong, China; ^3^Department of Radiology, Qujing Second People’s Hospital, Qujing, China

**Keywords:** 3D MERGE, CT-3D MERGE fusion imaging, image quality, lumbar disc herniation (LDH), fusion image, non contrast enhanced MRI

## Abstract

**Background:**

CT-routine MRI fusion imaging has recently become available to evaluate spinal anatomy before surgery. Due to the 3-5 mm slice thickness and non-isotropic of routine MRI sequence, the CT-routine MRI fusion imaging is not good. The MRI multiple recalled gradient echo (MERGE) sequence is potentially useful in diagnosis of lumbar degeneration disease due to the better nerve roots visualization, 1 mm slice thickness and its isotropy.

**Purpose:**

The present study aimed to evaluate the image quality of CT-3D MERGE fusion images compared with CT and 3D MERGE images in patients with lumbar disc herniation.

**Methods:**

Fifty-nine patients with lumbar disc herniation who underwent both lumbar CT and MRI including 3D-MERGE and routine lumbar MRI sequences were evaluated. All CT, 3D MERGE and CT-3D MERGE fusion images were separately assessed by two radiologists using five-point Likert scoring method based on five aspects: display of bony structure, intervertebral discs, nerve roots, overall anatomical details and image artifacts. Furthermore, two observers documented the sacral slope (SS), L4/5 intervertebral space heights (ISH), width and height of L4/5 intervertebral foramen (FW and FH) on CT and CT-MERGE fusion images.

**Results:**

There was insufficient evidence to show a difference in bony structure score between CT and CT-3D MERGE fusion images (*p* = 0.22), but it was significantly higher than that of MERGE (*p* < 0.001). The scores of intervertebral discs and nerve roots between MERGE and fusion images were not statistically different (*p* = 0.19 and 0.88), which were higher than CT (all *p* < 0.001). The overall anatomical detail score of fusion imaging was higher than CT and MERGE (*p* < 0.001). No significant difference of image artifacts score was found among CT, MERGE and fusion images (*p* = 0.47). There was no significant difference in SS, ISH, FW, FH values between CT and fusion images (all *p* > 0.05).

**Conclusion:**

CT-3D MERGE fusion images exhibit superior image quality to both CT and 3D MERGE for the simultaneous observation of bony structures, intervertebral discs, and nerve roots.

## Introduction

Lumbar disc herniation (LDH) is a common cause of low back pain and radiating pain to the lower limbs (1). The incidence of LHD was found to be approximately 5–20 cases per 1,000 adults annually in Norway (2). Surgical treatment could be required when patients with symptomatic LDH fail to improve with non-surgical care (3). Preoperative judgment of the location of lumbar disc herniation, nerve root compression, and bony spinal stenosis can affect the choice of surgical methods and prognosis. At present, the main methods of preoperative lumbar spine examination are computed tomography (CT) and magnetic resonance imaging (MRI) (4–6). CT examination can better display the bone anatomical structure, but the observation of spinal cord and nerve root is not good; MRI examination can clearly show the nerve root, spinal cord and intervertebral discs (7–9), but the anatomical structure of bone is not well displayed.

At present, the MRI multiple recalled gradient echo (MERGE) sequence has been applied to the imaging diagnosis of spinal cord in patients with multiple sclerosis, which can improve the detection and sensitivity of lesions in spinal cord (10–12). The MERGE MRI sequence is potentially useful in imaging of lumbar degeneration disease: the degenerated intervertebral discs and nerve roots showed hyper-intense on MERGE sequence while hypo-to iso-intense on T2-weighted fast spin echo (FSE) sequence. The MERGE sequence does not require the injection of contrast agents. In addition, isotropic MERGE sequence with thin thickness layer could achieve post-processing reconstruction. CT-routine MRI fusion imaging have recently become available to evaluate spinal anatomy before surgery (13–15), but there is no relevant study about fusion of CT and MRI MERGE sequence to observe the bony structure, intervertebral discs and nerve roots in patients with low back pain. The present study aimed to evaluate the image quality of CT-3D MERGE fusion imaging compared with CT and 3D MERGE sequence in patients with lumbar disc herniation.

## Materials and methods

This observational study was approved by the Medical Ethics Committee of Traditional Chinese Medicine (approval number: BF2022-181-01) and the requirement for informed consent was in line with the study protocol.

### Study population

Sixty-four patients suspected of lumbar disc herniation with lower back pain and radiating pain in the lower extremities were recruited between February and December 2023. All patients who underwent both CT and MRI in hospital were included in our study. Criteria for inclusion were: (1) age >18 years and <80 years; (2) patients have lower back pain accompanied by radiating pain in lower limb; and (3) no history of lumbar surgery. Five patients were excluded due to strong artifact. Finally, 59 patients were included, with 28 males and 31 females.

### CT and MR protocol

The imaging range was from the lower end of the T12 vertebra to the lower end of the S2 vertebra. CT scans were performed using a Canon 320-row detector CT device (Aquilion One Vision Edition; Canon Medical Systems, Otawara, Japan) with slice thickness1.0 mm, interval thickness 1.0 mm, tube voltage 120 kV and tube current 250 mA. From these data, the 3D spine image was reconstructed with slice thickness 1.0 mm.

All MR imaging studies were performed with a 3.0-T system (Discovery MR750 3.0 T with 32-channel GEM body coil; GE Healthcare). All 59 patients underwent lumbar MRI including coronal 3D-MERGE sequence and routine lumbar MRI sequences: T1-weighted and T2-weighted FSE sequence in sagittal and T2-weighted FSE axial planes. The MRI parameters were summarized in Table 1. CT and MRI scans try to ensure the consistency of patient position, as the changes of position could affect the accuracy of CT-3D MERGE image fusion.

**Table 1:** MERGE, T2-weighted FSE and T1-weighted FSE protocol parameters.

	MERGE	T2-weighted FSE	T1-weighted FSE	T2-weighted FSE
Repetition time (ms)	Minimum	2,609	599	5,988
Echo time (ms)	Min full	104	8	102
Imaging plane	Coronal	Sagittal	Sagittal	Axial
Flip angle	5°	142°	110°	142°
Slice gap (mm)	1.0	1.0	1.0	3.0
Slice thickness (mm)	1.0	4.0	4.0	4.0
Field of view	320*256	320*288	320*224	320*256
Number of excitations	6	6	3	6
Scan time	3:38	2:16	1:42	3:42

### Image analysis

The CT images and MERGE images both with 1.0 mm slice thickness and 1.0 mm slice gap were imported to GE workstation (AW VolumeShare 4.7; GE Healthcare) to obtain fusion images of CT - MRI 3D MERGE. The fusion images with 1.0 mm slice thickness were isotropic, thus the workstation automatically performed coronal, sagittal and axial three-dimensional reconstruction. The window width and window level of CT and CT-MERGE fused images can be adjusted to evaluate the image quality.

All CT, MR MERGE sequence and CT-3D MERGE fusion images were separately assessed by two radiologists (A and B, with 7 and 19 years of experience in musculoskeletal diagnosis). The image quality of CT, MERGE and fusion images were evaluated by two radiologists using double-blind five-point Likert scoring method from five aspects: display of bony structure, intervertebral discs, nerve roots, overall anatomical details (1 = unacceptable poorly display of above-mentioned anatomy details; 2 = poor display; 3 = moderate display; 4 = good display; 5 = excellent display) and image artifacts (1 = severe artifacts; 2 = moderate artifacts; 3 = mild artifacts; 4 = minimal artifacts; 5 = no artifacts). When the two radiologists had different opinions, they were to negotiate and reach a consensus. Intraclass correlation coefficients (ICCs) were used to assess inter-observer agreements for five-point Likert image quality score.

The sacral slope (SS) is the angle between the superior plate of S1 and a horizontal line. The degree of the sacral slope determines the position of the lumbar spine, since the sacral plateau forms the base of the spine (16, 17). For degenerated intervertebral discs, the intervertebral space height (ISH) was lower than normal discs. Two observers independently documented the sacral slope (SS), L_4/5_ ISH, width and height of L_4/5_ intervertebral foramen (FW and FH) on CT and CT-MERGE fusion images.

### Statistical analysis

Statistical analyses were performed using commercially available software SPSS 22.0 (SPSS, Chicago, IL, USA). Kruskal-Wallis test and follow-up unrelated Mann–Whitney test were used to detect differences in quality scores in bony structure, intervertebral discs, nerve roots, overall anatomical details display and image artifacts among CT, MERGE and CT-3D MERGE fusion images. Intraclass correlation coefficients (ICCs) were used to assess inter-observer agreements SS, ISH, FW and FH on CT and CT-3D MREGE fusion images (ICC: 0–0.20, poor correlation; 0.21–0.40, fair; 0.41–0.60, moderate; 0.61–0.80, good; and 0.81–1.00, excellent). The SS, ISH, FW, and FH values measured by two radiologists were averaged for further analysis. Paired-Samples t test was used to analyze the difference of SS, ISH, FW and FH between CT and CT-MERGE fusion images. In all tests, *p* < 0.05 was considered statistically significant.

## Results

The mean age of 59 patients was 58.31 ± 13.59 years (range, 29–79 years) in this study, including 28 males (mean age, 52.75 ± 12.26 years; range, 29–73 years) and 31 females (63.32 ± 12.93 years; range, 29–79 years).

**Figure 1:**
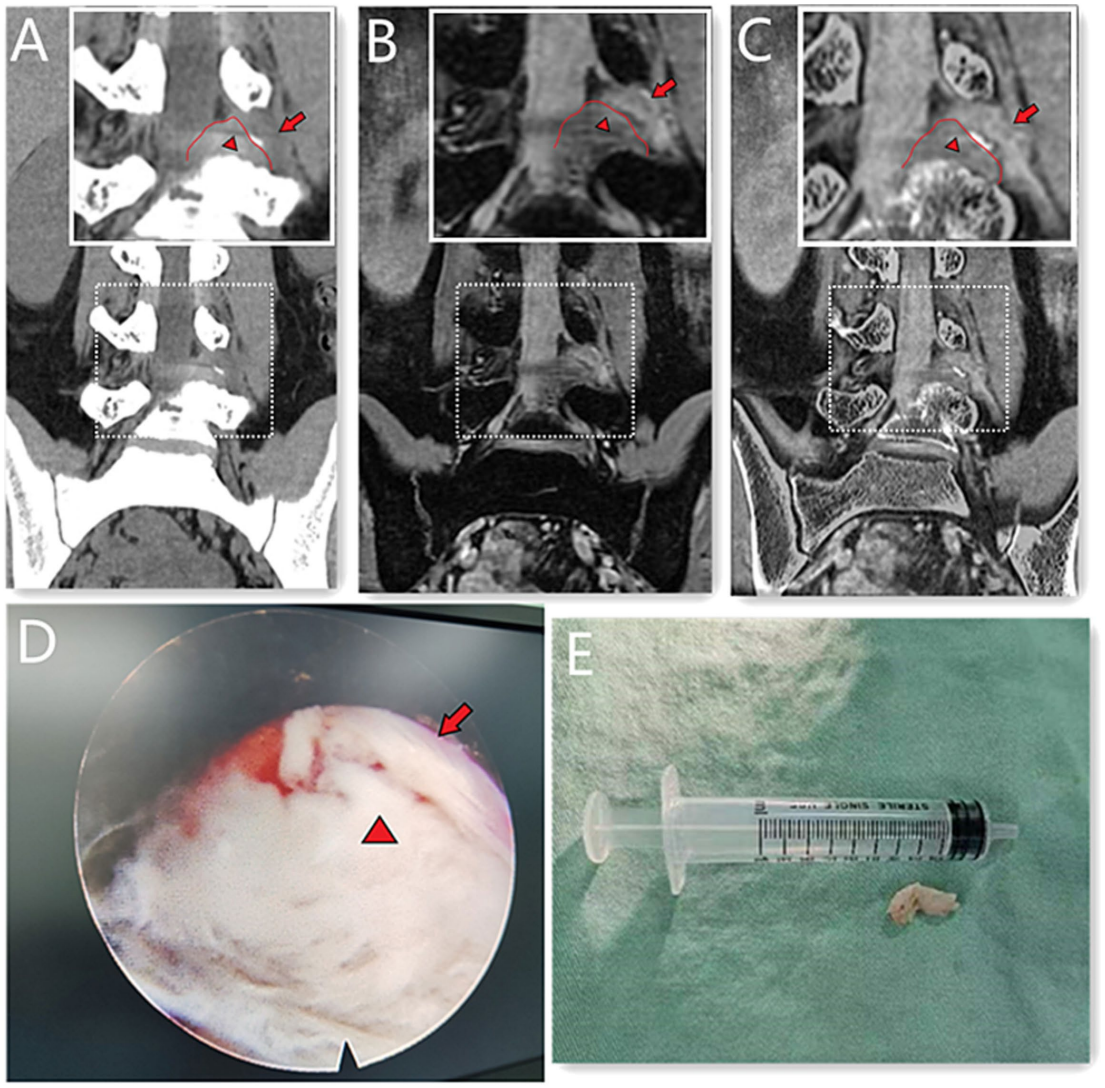
A 39-year-old male with L_4/5_ intervertebral disc herniation, compressing the left nerve root. The red lines indicate the edge of the herniated disc **(A–C)**. The arrow represents the nerve root and the arrow head represents the disc herniation **(A–E)**. The CT image **(A)** quality scores of bony structure, discs and nerve roots were 5^*^, 3, 3. The 3D MERGE **(B)** image quality scores were 2, 5 and 4. The image quality scores of CT 3D-MERGE fusion image **(C)** were 5, 5, and 4. **(D)** Showed a photo of transforaminal endoscopic lumbar discectomy for L_4/5_ and **(E)** showed the excised intervertebral disc. *The bone structure score of CT images was 5 points, which was evaluated in the bone window and not represented in figure.

**Figure 2:**
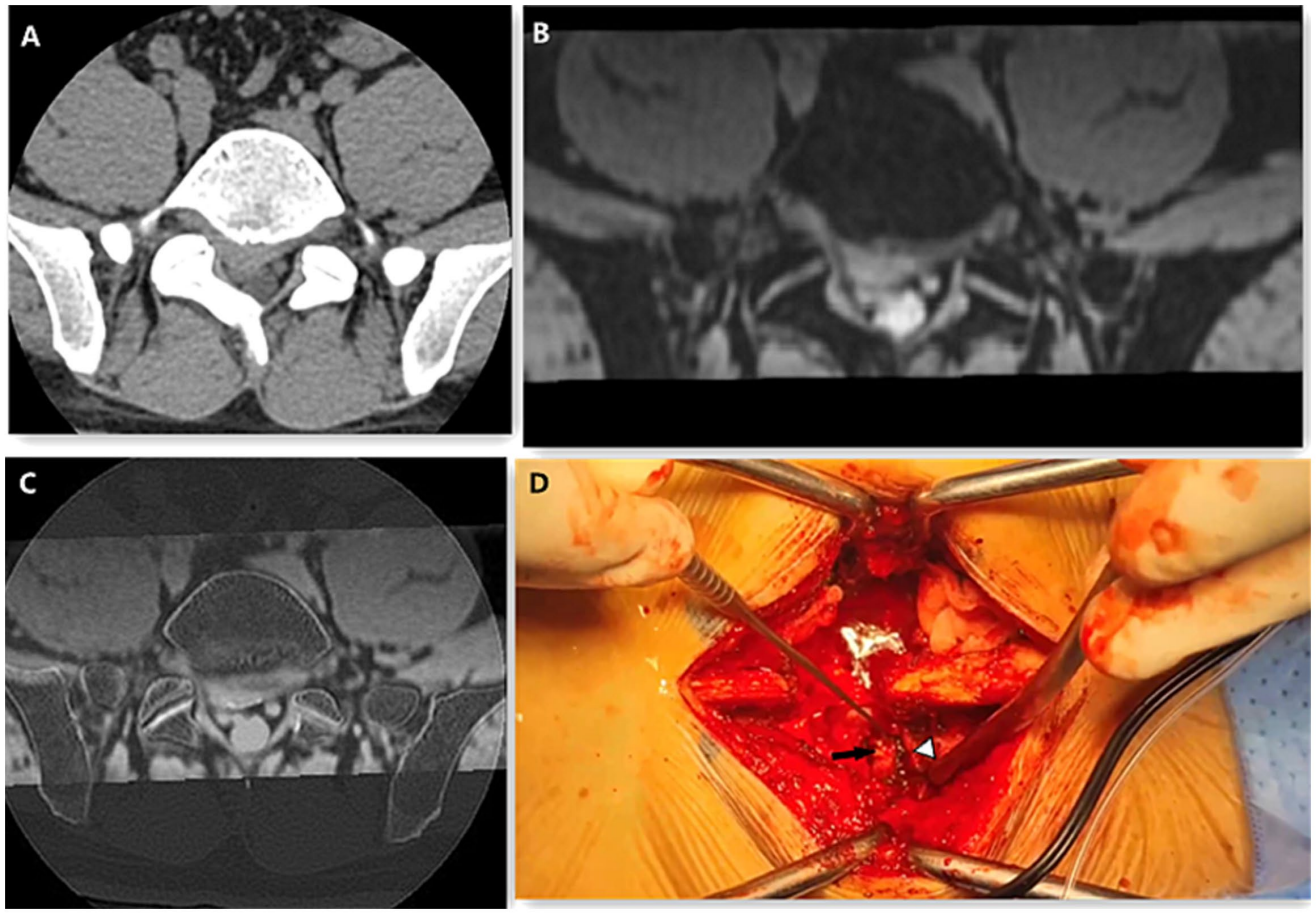
A 41-year-old male presented with L5/S1 disc herniation and right nerve root compression. The CT axial **(A)** image scores of bony structure, intervertebral disc and nerve root were 5, 4, 3. The scores of 3D MERGE **(B)** of bony structure, intervertebral disc and nerve root were 3, 5, 5. The image quality score of CT-3D MERGE fusion image **(C)** were 5, 5, 5. On the Photo of posterior lumbar interbody fusion surgery **(D)**, the black arrow shows the herniated disc and the white arrow head shows the nerve root.

The five-point Likert scores of CT, 3D MERGE MRI and CT-3D MERGE fusion images were shown in Table 2. The ICCs for five-point Likert scores were excellent agreement (all>0.95) between two radiologists. No sufficient evidence showed a difference in bony structure score between CT and CT-3D MERGE fusion images (*p* = 0.22), but it was significantly higher than that of MERGE (*p* < 0.001) (Figures 1, 2). The quality scores of intervertebral discs and nerve roots between 3D MERGE and fusion images were not statistically different (*p* = 0.19 and 0.88), which were higher than that of CT (all *p* < 0.001) (Figures 1, 2). For the score of overall anatomical details, CT-3D MERGE fusion images was higher than that of CT and MERGE (all *p* < 0.001). There’s no significant difference in image artifacts score among CT, MERGE and CT-3D MERGE images (*p* = 0.47).

**Table 2:** The Likert scores of CT-3D MERGE fusion images, CT and 3D MERGE sequence.

	CT-3D MERGE	CT	3D MERGE	*p* value
Bony structure	5 [5, 5]	5 [5, 5]	3 [3, 4]	<0.001
Intervertebral discs	5 [5, 5]	4 [3, 4]	5 [4, 5]	<0.001
Nerve roots	5 [5, 5]	3 [3, 4]	5 [5, 5]	<0.001
Overall anatomical details	5 [5, 5]	4 [4, 4]	4 [4, 4]	<0.001
Image artifacts	5 [5, 5]	5 [5, 5]	5 [5, 5]	0.47

For both observers, there were excellent intra-class correlation agreements in values of SS, ISH, FW and FH between CT and fusion images (all>0.90). There was no significant difference in SS, ISH, FW, FH values between CT and CT-3D MERGE fusion images (all *p* > 0.05) (Table 3).

**Table 3:** The SS, ISH, FW, FH values between CT-MERGE and CT.

	CT-MERGE	CT	*p* value
SS	37.29 ± 7.66	37.27 ± 7.71	0.88
ISH	10.74 ± 2.70	10.81 ± 2.71	0.20
FW	10.74 ± 1.89	10.70 ± 1.92	0.26
FH	18.05 ± 2.48	17.95 ± 2/47	0.14

SS, Sacral slope; ISH, Intervertebral space height; FW, Width of intervertebral foramen; FH, Height of intervertebral foramen.

## Discussion

This study compared image quality using CT, 3D MERGE and CT-3D MERGE fusion images in lumbar herniation patients. CT-3D MERGE fusion images had the best image quality scores in bony structure, discs, nerve roots and overall anatomical details. The bony structure score of CT-3D MERGE fusion images showed no significant difference from CT, which was higher than MERGE. There was insufficient evidence to show a difference in quality scores of intervertebral discs and nerve roots between fusion images and 3D MERGE MRI, but both were significantly higher than that of CT. The fusion images had the highest overall anatomical detail score. In addition, there was no statistically significant difference in the SS, ISH, FW, and FH values between CT-3D MERGE and CT images, further demonstrating the accuracy of fusion images in measuring bony structures.

CT is an effective non-invasive examination. The density value (Hounsfield unit, HU) of CT depends on the mass density of the material and the degree of X-ray attenuation. Therefore, osseous tissue, especially the cortical bone, appears high-density and “white” on CT, highlighting the bony tissue. However, unlike MRI offers unparalleled soft tissue contrast, CT has limited ability to provide soft tissue detail (18, 19), and the anatomical details of intervertebral discs and nerve roots with iso-density are not well displayed. On T1 and T2 weighted images, low signal from nerve are surrounded by high signal from fat in the intervertebral foramen. T2 weighted imaging shows decreased signal of degenerative intervertebral discs. Both the nerve roots and the degenerated intervertebral discs are hyper-intensities on 3D MERGE MRI, which is more distinct. Cortical bone appears as a structure with low signal intensity that is not specific to bone in conventional MRI (20), as well as 3D MERGE sequence. In CT-3D MERGE fusion images, the bony structure, especially bone cortex showed high density (“white”), and both degenerated discs and nerve roots showed hyper signal intensity. This provides more accurate diagnostic information for us to judge the relationship of the above three parts simultaneously. As a result, the image quality of CT-3D MERGE fusion images was higher than CT and 3D MERGE MRI sequence.

T2-weighted three dimensional FSE with fat suppression technology sequence is mostly used for lumbosacral plexus imaging (21–23). This sequence is named differently by various vendors as CUBE (GE Healthcare), SPACE (Siemens), and VISTA (Philips). However, these T2-weighted 3D FSE techniques have some drawbacks, including longer acquisition time compared to MERGE, degenerated discs with hypo-to iso-intensity that making it difficult to determine the relationship between intervertebral disc margin and nerve roots, and the hyperintense artifacts caused by vascular structures alongside nerve, leading to difficulties in nerve visualization (22, 23). As we know, GRE often suffers from limited gray matter (GM)/white matter (WM) contrast and contrast to better visualize nerve roots and foraminal stenoses. The 3D MERGE sequence combines multiple bipolar gradient-echo formations using early echoes to increase SNR and later echoes to increase image contrast, improving GM/WM contrast and nerve roots visualization (24). This sequence is known by a variety names depending on the vendors: MERGE (GE Healthcare), MEDIC (Siemens) and MFFE (Philips). To date, this sequence has most commonly been used to detect of multiple sclerosis lesions in spinal cord (10–12) and evaluation of cartilage (25–27). In addition, unlike on spin echo (SE) and FSE sequences, the degenerated discs (hyperintense) and osteophytes (hypointense) could usually be differentiated with 2D or 3D GRE regardless of flip angle (28). The MERGE sequence has high resolution and isotropy, which could better observe the relationship between nerve root and discs after three-dimensional reconstruction. Therefore, the CT-3D MERGE fusion imaging combines the advantages of both CT and 3D MERGE sequence. By adjusting the window width and level of CT and performing reconstruction, the fusion imaging could obtain better image quality.

Our study has some limitations. First, the sample size was small. Second, we were not able to compare MERGE with CUBE sequences, as well as CT-MERGE with CT-CUBE fusion images, as the CUBE sequence was not included in our routine lumbar MRI scan. Patients suffer from low back pain may not endure longer scanning time. Moreover, the image quality was evaluated only by radiologists and surgeons were not involved. Further study is needed to compare MERGE with CUBE and orthopedic spine surgeons will participate in evaluating image quality of different fusion images.

## Conclusion

CT-3D MERGE fusion images exhibit superior image quality to both CT and 3D MERGE for simultaneously observing bony structures, intervertebral discs, and nerve roots.

## Data Availability

The raw data supporting the conclusions of this article will be made available by the authors, without undue reservation.

## References

[1] Campbell P Wynne-Jones G Muller S Dunn KM. The influence of employment social support for risk and prognosis in nonspecific back pain: a systematic review and critical synthesis. Int Arch Occup Environ Health. (2013) 86:119–37. doi: 10.1007/s00420-012-0804-2, PMID: 22875173 PMC3555241

[2] Pojskic M Bisson E Oertel J Takami T Zygourakis C Costa F. Lumbar disc herniation: epidemiology, clinical and radiologic diagnosis WFNS spine committee recommendations. World Neurosurgery X. (2024) 22:100279. doi: 10.1016/j.wnsx.2024.100279, PMID: 38440379 PMC10911853

[3] Yoon WW Koch J. Herniated discs: when is surgery necessary? EFORT Open Rev. (2021) 6:526–30. doi: 10.1302/2058-5241.6.210020, PMID: 34267943 PMC8246101

[4] Haughton V. Medical imaging of intervertebral disc degeneration: current status of imaging. Spine. (2004) 29:2751–6. doi: 10.1097/01.brs.0000148475.04738.73 15564924

[5] D’Aprile P Tarantino A Jinkins JR Brindicci D. The value of fat saturation sequences and contrast medium administration in MRI of degenerative disease of the posterior/perispinal elements of the lumbosacral spine. Eur Radiol. (2007) 17:523–31. doi: 10.1007/s00330-006-0324-0, PMID: 16733673

[6] D’Aprile P Nasuto M Tarantino A Cornacchia S Guglielmi G Jinkins JR. Magnetic resonance imaging in degenerative disease of the lumbar spine: fat saturation technique and contrast medium. Acta Bio Medica. (2018) 89:208–19. doi: 10.23750/abm.v89i1-S.7024 PMC6179082 29350649

[7] Hasegawa T Mikawa Y Watanabe R An HS. Morphometric analysis of the lumbosacral nerve roots and dorsal root ganglia by magnetic resonance imaging. Spine. (1996) 21:1005–9. doi: 10.1097/00007632-199605010-00001, PMID: 8724082

[8] Adams A Roche O Mazumder A Davagnanam I Mankad K. Imaging of degenerative lumbar intervertebral discs; linking anatomy, pathology and imaging. Postgrad Med J. (2014) 90:511–9. doi: 10.1136/postgradmedj-2013-132193 24965489

[9] Simon J McAuliffe M Shamim F Vuong N Tahaei A. Discogenic low back pain. Phys Med Rehabil Clin N Am. (2014) 25:305–17. doi: 10.1016/j.pmr.2014.01.006 24787335

[10] Ozturk A Aygun N Smith SA Caffo B Calabresi PA Reich DS. Axial 3D gradient-echo imaging for improved multiple sclerosis lesion detection in the cervical spinal cord at 3T. Neuroradiology. (2012) 55:431–9. doi: 10.1007/s00234-012-1118-5, PMID: 23208410 PMC3602327

[11] White ML Zhang Y Healey K. Cervical spinal cord multiple sclerosis: evaluation with 2D multi-echo recombined gradient echo MR imaging. J Spinal Cord Med. (2013) 34:93–8. doi: 10.1179/107902610X12911165975025 PMC3066479 21528632

[12] Martin N Malfair D Zhao Y Li D Traboulsee A Lang D. Comparison of MERGE and axial T2-weighted fast spin-Echo sequences for detection of multiple sclerosis lesions in the cervical spinal cord. Am J Roentgenol. (2012) 199:157–62. doi: 10.2214/AJR.11.7039, PMID: 22733907

[13] Nagamatsu M Ruparel S Tanaka M Fujiwara Y Uotani K Arataki S. Assessment of 3D lumbosacral vascular anatomy for OLIF51 by non-enhanced MRI and CT medical image fusion technique. Diagnostics. (2021) 11:1744. doi: 10.3390/diagnostics11101744, PMID: 34679442 PMC8534854

[14] Nagamatsu M Maste P Tanaka M Fujiwara Y Arataki S Yamauchi T. Usefulness of 3D CT/MRI fusion imaging for the evaluation of lumbar disc herniation and Kambin’s triangle. Diagnostics. (2022) 12:956. doi: 10.3390/diagnostics12040956, PMID: 35454004 PMC9031438

[15] Aoyama R Anazawa U Hotta H Watanabe I Takahashi Y Matsumoto S. The utility of augmented reality in spinal decompression surgery using CT/MRI fusion image. Cureus. (2021) 13:e18187. doi: 10.7759/cureus.18187, PMID: 34589373 PMC8459800

[16] Legaye J Duval-Beaupère G Hecquet J Marty C. Pelvic incidence: a fundamental pelvic parameter for three-dimensional regulation of spinal sagittal curves. Eur Spine J. (1998) 7:99–103. doi: 10.1007/s005860050038, PMID: 9629932 PMC3611230

[17] Berthonnaud E Labelle H Roussouly P Grimard G Vaz G Dimnet J. A variability study of computerized sagittal spinopelvic radiologic measurements of trunk balance. J Spinal Disord Tech. (2005) 18:66–71. doi: 10.1097/01.bsd.0000128345.32521.43, PMID: 15687855

[18] Nam D Barrack RL Potter HG. What are the advantages and disadvantages of imaging modalities to diagnose wear-related corrosion problems? Clin Orthop Relat Res. (2014) 472:3665–73. doi: 10.1007/s11999-014-3579-9, PMID: 24664197 PMC4397750

[19] Attenberger UI Morelli J Budjan J Henzler T Sourbron S Bock M. Fifty years of technological innovation: potential and limitations of current Technologies in Abdominal Magnetic Resonance Imaging and Computed Tomography. Investig Radiol. (2015) 50:584–93. doi: 10.1097/RLI.0000000000000173, PMID: 26039773

[20] Florkow MC Willemsen K. Magnetic resonance imaging versus computed tomography for three‐dimensional bone imaging of musculoskeletal pathologies: a review. J Magn Reson Imaging. (2022) 56:11–34. doi: 10.1002/jmri.28067 35044717 PMC9305220

[21] Cervantes B Bauer JS Zibold F Kooijman H Settles M Haase A. Imaging of the lumbar plexus: optimized refocusing flip angle train design for 3D TSE. J Magnetic Resonance Imaging. (2016) 43:789–99. doi: 10.1002/jmri.25076, PMID: 26454005

[22] Chhabra A Rozen S Scott K. Three-dimensional MR neurography of the lumbosacral plexus. Semin Musculoskelet Radiol. (2015) 19:149–59. doi: 10.1055/s-0035-1545077, PMID: 25764239

[23] Delaney H Bencardino J Rosenberg ZS. Magnetic resonance neurography of the pelvis and lumbosacral plexus. Neuroimaging Clin N Am. (2014) 24:127–50. doi: 10.1016/j.nic.2013.03.026, PMID: 24210317

[24] Vertinsky AT Krasnokutsky MV Augustin M Bammer R. Cutting-edge imaging of the spine. Neuroimaging Clin N Am. (2007) 17:117–36. doi: 10.1016/j.nic.2007.01.003, PMID: 17493543 PMC2080848

[25] Higashihira S Kobayashi N Oishi T Choe H Ike H Tezuka T. Comparison between 3-dimensional multiple-Echo recombined gradient Echo magnetic resonance imaging and arthroscopic findings for the evaluation of acetabular labrum tear. Arthroscopy. (2019) 35:2857–65. doi: 10.1016/j.arthro.2019.05.006, PMID: 31604505

[26] Aydın N Saylısoy S Adapınar B Arslantas D. A comparative evaluation of the Eustachian tube cartilage between healthy and diseased ears using a 3 tesla MRI. Pol J Radiol. (2020) 85:e581–5. doi: 10.5114/pjr.2020.99756, PMID: 33204372 PMC7654315

[27] Nardo L Carballido-Gamio J Tang S Lai A Krug R. Quantitative assessment of morphology, T(1ρ), and T(2) of shoulder cartilage using MRI. Eur Radiol. (2016) 26:4656–63. doi: 10.1007/s00330-016-4322-6, PMID: 26993651 PMC5027178

[28] Yousem DM Atlas SW Goldberg HI Grossman RI. Degenerative narrowing of the cervical spine neural foramina: evaluation with high-resolution 3DFT gradient-echo MR imaging. AJNR Am J Neuroradiol. (1991) 12:229–36. PMID: 1902018 PMC8331411

